# Latent Autoimmune Diabetes Mellitus in Adults (LADA) and it’s characteristics in a subset of Nigerians initially managed for type 2 diabetes

**DOI:** 10.1186/1755-7682-5-23

**Published:** 2012-08-15

**Authors:** Olufunmilayo O Adeleye, Anthonia O Ogbera, Olufemi Fasanmade, Olayinka O Ogunleye, Akinola O Dada, Ayotunde O Ale, Femi M Abatan

**Affiliations:** 1 Department of Medicine, Lagos State University Teaching Hospital, 1-5 Oba Akinjobi Way, Ikeja, Lagos, Nigeria; 2 Department of Medicine, Lagos University Teaching Hospital, Idi Araba, Lagos, Nigeria; 3 Department of Chemical Pathology, Lagos University Teaching Hospital, Idi-Araba, Lagos, Nigeria

**Keywords:** LADA, Prevalence, Complications, Nigerians

## Abstract

**Introduction:**

Latent autoimmune diabetes in adults (LADA) is an entity characterized by the presence of GAD autoantibodies. LADA is largely understudied and underreported amongst Nigerians with Diabetes Mellitus (DM). We undertook to document the Prevalence, clinical and biochemical characteristics of LADA in a subset of Nigerians who hitherto had been treated for type 2 DM.

**Methods:**

This is a cross-sectional study conducted on 235 patients being managed for type 2 DM. The diagnosis of LADA was made in the presence of Glutamic Acid Decarboxylase autoantibody (GADA) positivity in the study subjects. Thereafter persons with LADA were compared with those without LADA. Clinical parameters such as demographic data, history of diabetes mellitus (DM) and its complications were obtained, biochemical parameters including Fasting blood glucose (FBG), C-peptide, glycated haemoglobin (HbA_1c_) and lipid parameters were compared in both groups of Study subject. Test statistics used were Student t- test and χ 2. SPSS was used for data analysis.

**Results:**

Thirty three out of 235 of the Study subjects were GADA positive, giving a prevalence of 14%. The mean age (SD) of the subjects with LADA is 53.24(7.22) with an age range of 30–63 years. Majority (48%) of LADA subjects were in the 50–59 age category. There was no significant difference in the proportion of males and females with LADA (*p =* 0.3). 37% of patients with LADA were on insulin for glycaemic control. Three (3) LADA subjects had history/clinical evidence of autoimmune thyroid disease. 66% of LADA were in the overweight/obese category. LADA subjects had significant poor long term glycaemic control compared with anti-GAD negative subjects (*p =* 0.026). About half of LADA subjects were insulinopaenic. LADA subjects had lower levels of total cholesterol than GADA-ve subjects (*p =* 0.03). A higher proportion of LADA had evidence of microvascular complications of DM compared with antiGAD negative individuals.

**Conclusion:**

The diagnosis of LADA should be entertained in overweight/obese persons from the fourth decade of life presenting with DM. Pharmacotherapy with insulin is a potential means of managing hyperglycaemia in this group of patients especially since a significant proportion are insulinopaenic. The Prevalence of LADA in our patients is comparable to what obtains in Ghanaian and Caucasian populations.

## Introduction

Latent Autoimmune Diabetes in Adults (LADA) was first identified in a subset of phenotypic type 2 diabetic individuals who were positive for islet cell antibodies and failed to respond to sulphonylurea therapy [1]. LADA is used to describe a form of autoimmune diabetes that has a later onset and a slower progression toward an absolute insulin requirement [2]. The presence of pancreatic auto antibodies especially to Glutamic acid decarboxylase (GAD65) is the best single marker required for screening for LADA [2,3]. Although various cut –off ages have been used in previous studies on LADA, the current proposed lower limit is 30 years [2]. Besides the acronym-LADA, this subset of individuals have also been named “type 1.5 diabetes”, “slowly progressive type 1 diabetes”, “latent type 1 diabetes”, “youth onset diabetes of maturity”, “LADA type1” and “LADA type2”[4].

The presence of LADA has been reported worldwide with documented regional differences in the prevalence of LADA. These variations in the estimated prevalences may be due to the application of non-uniform criteria in the diagnosis of LADA. The increasing prevalence of type 2 DM in some populations could also influence the prevalence of LADA. The United Kingdom Prospective Diabetes Study [5] detected antiGAD autoantibody positivity in 10% of people studied while a population based study in Wales found that 4% of people with phenotypic features of type 2 diabetes were antiGAD autoantibody positive with an incidence of LADA of 9 per 100,000 people/year [6].

Reports from Korea indicated a prevalence of LADA between 5.1%–5.3% in population based studies [7,8]. Report from China indicated LADA prevalence of 9.2% in a population of phenotypic type 2 DM patients who had hypertension, family history of diabetes and central obesity [9].

A Ghanaian study reported a prevalence of LADA of 13.5% in non-insulin requiring diabetic patients [10]. [Oli et al 11] in 1981 measured pancreatic islet autoantibodies in 68 Nigerians with type 2 DM, and only 4 subjects were found to express significant titers of antiGAD.

Evidence obtained from studies, indicate that preservation of β cell function in patients with autoimmune diabetes results in easier and better glycaemic control and fewer end organ complications especially retinopathy, hence the need for proper classification of diabetic subjects. We therefore set out to determine the prevalence of LADA, the clinical characteristics, disease burden and compare same with GAD negative type 2 diabetic subjects.

## Methods

This is a descriptive hospital based study. The study was carried out at the Diabetes Center of the Lagos State University Teaching Hospital (LASUTH) located in Ikeja Lagos. LASUTH is a state owned tertiary health facility that receives referrals from parts of Western Nigeria.

### Subjects

Two hundred and thirty five subjects (235) with Type 2 DM [12] who satisfied the inclusion criteria and gave consent were selected by systematic random sampling (every 5^th^ consecutive patient recruited out of the total number of type 2 DM patients attending the outpatient clinic for follow up each clinic day. LASUTH diabetes center runs two clinics/week with an average of 80 diabetic patients seen each clinic day) for antiGAD autoantibody (GADA) studies. The study was conducted over duration of 6 months.

Inclusion criterion was individual with type 2 DM diagnosis at age of 30 years and above.

Pregnant women, patients taking steroid medications and those with cancer and severe co-morbidities were excluded.

The study was approved by the Research and Ethics Committee of LASUTH.

Standardized questionnaire(s) administered by qualified physician and patients’ hospital records were employed to collect participants’ demographic and clinical data including age, sex, disease duration, family history of DM, whether on insulin for glycaemic control, duration of insulin therapy, history of other organ specific autoimmune diseases, history of and duration of hypertension, dyslipidaemia and DM complications including retinopathy, neuropathy, nephropathy and DM foot syndrome.

Venous blood samples were obtained for Antiglutamic acid decarboxylase autoantibodies, sera were separated within one hour and refrigerated at -20°C.

The second phase of the study involved individuals who were GADAs positive and age, sex, duration of DM matched GADAs negative controls. (I.e. A sample of the 202 GADAs negative whose ages, gender and disease duration were similar to the 33 GADAs positive subjects) Overnight fasting venous blood was obtained for plasma glucose, lipids, C-peptide and glycosylated hemoglobin (HbA_1_C). Urine passed into universal bottles was assessed for macro and microalbuminuria using the Uripath 10 urinalysis test strip (Plasmatic labs. United Kingdom) and Micral test strips (Roche Diagnostics Australia) respectively.

Clinical examination done on second phase of study includes standardized anthropometric indices. i.e The weight of each individual was measured using a weighing scale, (metric weighing scale by Avery Co, England.) with the subject standing still in the center of the weighing scale’s platform, wearing light clothing. Weight was recorded to the nearest 0.5 kg [13], the height was measured with a stadiometer and recorded to nearest 0.1 cm. Waist circumference was measured using a metric non-stretching measuring tape, midway between the inferior margin of the lowest rib and the iliac crest in the horizontal plane, at the end of normal expiration. The hip circumference was measured using the same tape at the largest circumference of the gluteal region. Blood pressure (BP) was measured in the supine and upright positions [14], Presence of microvascular complications of retinopathy, nephropathy and neuropathy were assessed.

### Biothesiometry

Lowest voltage at which vibration is perceived in both feet was recorded.

### Laboratory assessment

#### Glutamic acid decarboxylase autoantibody

The laboratory (labs) determination of antigad autoantibodies was done at Immunoassay labs, the pioneer Labs for Enzyme Linked Immunosorbent Assay (ELISA) and Radioimmunoassay technique in Nigeria.

Serum antiGAD autoantibody levels were determined by ELISA technique using the RSR immunoprecipitation ELISA assay method (RSR Ltd. Cardiff, Wales) in which Elisa plate wells are coated with GAD and after incubation with test sera, antibodies monovalent bound to the coated wells are detected by addition of GAD biotin.

Cut-off limit of detection of antiGAD autoantibody levels is <5units/ml; Positive ≥ 5units/ml.

#### Fasting plasma glucose (FPG)

Plasma glucose concentration was measured using a glucose oxidase method [15].

#### C-peptide

Serum C-peptide levels were determined by an ELISA technique using the DAI C-peptide assay method (Diagnostic Automation System CA. USA). DAI C-peptide Quantitative test system is a solid phase Enzyme-linked Immunosorbent Assay.

#### Glycosylated hemoglobin (HbA1c %)

HbA1c was assayed using a fully automated Boronate Affinity assay for the determination of the percentage of hemoglobin A1C (HbA1c %) in whole blood.

#### Lipids

Total cholesterol assay was done using a modified method of [Liebermann-Burchard 16], TG was estimated with a kit using enzymatic hydrolysis of TG with lipases [17], and HDL-c was estimated by precipitation method [18]. LDL-c calculation was done using Friedwald’s Formula [19] LDL-c = (TC – HDL-c) – TG/5 when the values of TG were less than 400 mg/dl.

#### Operational definitions

1. LADA is said to be present if an individual diagnosed with DM above 30 years of age is antiGAD autoantibody positive and requires insulin for glycaemic control within six months of diagnosis [2].

2. Normal fasting blood glucose <6.1mmo/l or 110 mg/dl [12].

3. Type 2 DM: Individual with hyperglycemia diagnosed by fasting plasma glucose ≥7.0 mmol/l(126 mg/dl) and/2 hours post glucose load ≥ 11.1 mmol (200 mg/dl) on a single occasion in the presence of classic symptoms of diabetes or confirmed on a repeat occasion in the absence of symptoms [12].

4. Hypertension: Individuals with raised arterial pressure ≥140/90 or patients on antihypertensive medication [14].

5. Weight category [20].

a. Normal weight is taken as- BMI 18.5–24.9 kg/m^2^

b. Overweight- BMI 25.0–29.9 kg/m^2^

c. Obesity- BMI ≥ 30 kg/m^2^

6. Centripetal obesity is present if the waist circumference is above 94 cm in males and above 80 cm in females [21].

7. Metabolic syndrome: Individuals who satisfy the International Diabetes Federation (IDF) criteria for the metabolic syndrome [21] which include: Central obesity (defined as waist circumference ≥ 94 cm for men and ≥ 80 cm for women.

Plus any two of the following four factors:

Raised Triglyceride (TG) level ≥ 150 mg/dl (1.7 mmol/l), reduced High Density Lipoprotein cholesterol (HDL-c) <40 mg/dl (1.03 mmol/l) in males and <50 mg/dl (1.29 mmol/l) in females, raised blood pressure (BP): systolic BP ≥130 or diastolic BP ≥85 mmHg, -raised fasting plasma glucose (FPG) ≥ 100 mg/dl (5.6 mmol/l) or previously diagnosed type 2 diabetes.

8. Glycosylated hemoglobin (HbA1c). Glycaemic control is considered optimal if HbA1c is <7% [22]

9. C-peptide level of lower than 0.3 nmol/l indicates insulinopaenia [23]

10. DM Neuropathy refers to abnormal values based on digital biothesiometer values [24].

In subjects up to 50 years of age;

In subjects above 50 years;

Below 10 volts-Normal

Between 11 and 15 volts-Mild Neuropathy

Between 16 and 20 volts-Moderate Neuropathy

Above 21 volts – Severe Neuropathy

Below 15 volts-Normal

Between 16 and 20 volts- Mild Neuropathy

Between 21 and 25 volts- Moderate Neuropathy

Above 25 volts- Severe Neuropathy.

11. DM retinopathy refers to clinical evidence of background retinopathy or proliferative diabetic retinopathy on fundoscopy [25].

12. DM nephropathy refers to proteinuria (micro or macroalbuminuria) in the setting of diabetes without symptoms of urinary tract infection [26].

13. Lipid abnormalities refers to biochemical evidence of elevated values of total Cholesterol ≥ 200 mg/dl, HDL-c for men ≤ 40 mg/dl and women ≤ 50 mg/dl, low density lipoprotein(LDL-c) ≥ 100 mg/dl, and triglycerides ≥ 150 mg/dl, either singly or in combination [21,27].

14. Disease burden in this context refers to the prevalence of LADA, the occurrence of retinopathy, nephropathy, neuropathy, dyslipidaemia and the metabolic syndrome.

### Analyses and statistical evaluation of data

Calculation and analysis were done using Statistical Package for Social Science (SPSS for windows version 17. SPSS institute Chicago IL. USA). Quantitative data were expressed as mean ± standard deviation (SD) while qualitative data are expressed as percentages. Test statistics used include student *t*-test for quantitative data and χ 2 for qualitative data. A p-value of < 0.05 is regarded as significant.

## Results

The mean age (SD) of the study subjects is 51.9(8.5) with age range of 30–69 years. Sixty seven percent were females while males made up 33% of the study subjects Table 1.

**Table 1:** Pattern of clinical history of subjects with LADA

**CHARACTERISTICS**	**FREQUENCY**	**PERCENTAGE (%)**
Family hx of DM	13	39
Insulin usage	11	37
Thyroid disease (graves’)	3	11
History of hypertension	16	49
History of Dyslipidaemia	9	30
History of foot ulcer	1	4
History of stroke	1	4
History of significant alcohol use	1	4
History of smoking	1	4

*Hx*, History. *DM*, diabetes mellitus.

Table 1 shows the pattern of clinical history of subjects with LADA.

Out of the 235 patients studied 33(14%) were antiGAD autoantibody positive which gives a prevalence of LADA of 14%. 15% of male subjects were antiGAD positive and 13% of female subjects were antiGAD positive but the difference was not statistically significant *p* = 0.63.

**Figure 1:**
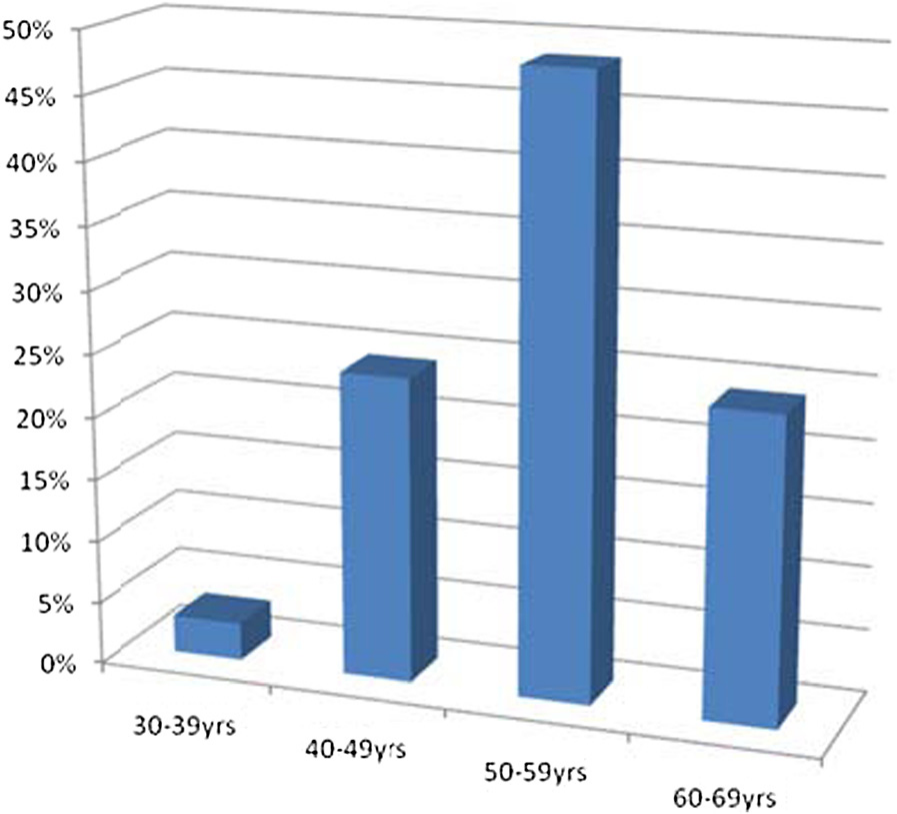
Prevalence of LADA by age categories.

The mean age (SD) of the subjects with LADA is 53.24(7.22) with an age range of 30–63 years. The prevalence of LADA according to different age categories is shown in Figure 1.

**Figure 2:**
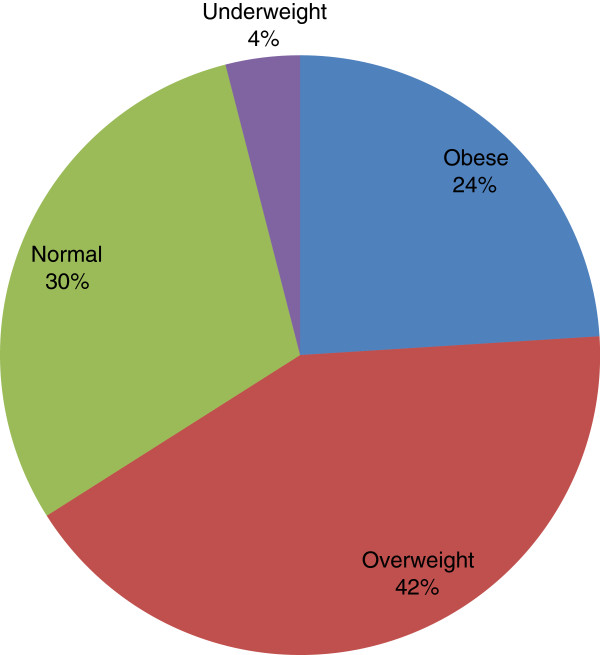
Pattern of Body Mass Indices of subjects with LADA.

Pattern of body mass indices of subjects with LADA is shown in Figure 2.

The mean (SD) glycosylated Hemoglobin (HbA1c) was 8.4(1.8) % with range of 5–12%. An analysis for glycemic control showed that 33% had good short term glycaemic and 26% had good long term glycemic control.

41% of the LADA subjects had the fasting C-peptide values of ≤ 0.3 nmol/dl.

The metabolic syndrome was noted in 45% of the LADA subjects.

### Comparison of LADA with GADAs negative

The mean age (SD) of LADA and GADA-ve subjects was 53.2(7.2) and 51.7(8.7).

The presence of autoimmune diseases specifically autoimmune thyroid disease (AITD) was noted in 3 (11%) of the subjects with LADA. AITD was absent in GADA negative subjects.

The proportion of subjects with LADA on sole insulin therapy was higher than those who are GADA negative, 37% vs 19%, *p =* 0.06.

The proportion of subjects with LADA who had history of hypertension was comparable to that of GADA-ve subjects who had history of hypertension (58% vs 59%, *p =* 0.9).

All studied anthropometric indices and some clinical parameters were comparable in subjects with LADA and GADA negative subjects Table 2.

**Table 2:** Clinical characteristics of LADA compared with GADA-ve subjects

**Variable**	**LADA**	**GADA-ve**	**p-value**
	**Mean(SD)**	**Mean(SD)**	
Duratn of DM (years)	6.43 (4.81)	7.1 (6.8)	0.30
BMI (kg/m2)	27.05 (6.54)	27.92 (5.3)	0.71
WC (cm)	90.90 (15.38)	94.5 (13.7)	0.71
Waist/hip	0.91 (0.08)	0.92 (0.06)	0.09
Systolic BP (mmHg)	142.96 (22.15)	146.8 (27.8)	0.17
Diastolic BP (mmHg)	88.14 (11.46)	87.75 (15.15)	0.90

*BMI*, Body Mass Indices, *WC*,Waist Circumferences, *BP* Blood Pressure, Autoantibody –ve, Autoantibody negative*, p* <0.05 significant.

Table 2 depicts comparison of clinical characteristics of LADA and GADA negative subjects.

On the assessment of β-cell function, 41% of LADA were insulinopenic with fasting c-peptide lower than 0.3 nmol/l. In contrast, 7% of GADA –ve were insulinopenic (*p* = 0.021).

A lower proportion of LADA had good long term (long term glycaemic control assessed with HbA1c) glycaemic control compared with GADA-ve, 26% vs 45% respectively and this was statistically significant, *p* =0.026. Indices of short term glycaemic control were however comparable between the two groups, *p* = 0.65.

**Figure 3:**
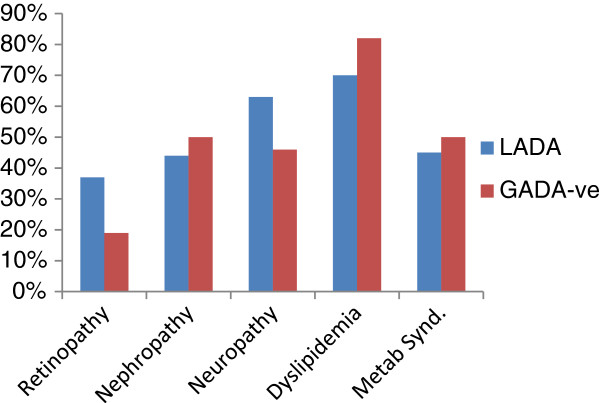
Comparison of Microvascular Complications, Dyslipidemia and Metabolic syndrome.

The prevalence of microvascular complications in LADA and GADA-ve individuals were comparable, 37% and 19% retinopathy, 44% and 50% nephropathy, 63% and 42% neuropathy with the respective p-values of 0.11, 0.6, and 0.3. These results and those of the metabolic syndrome in both groups of the study subjects are shown in Figure 3.

## Discussion

The prevalence of LADA in this report was found to be 14%. This finding is comparable to previous reports from Ghana where documented prevalence rates for LADA amongst people being managed for type 2 DM was 13.5% [10]. [Lutale et al 28] found low prevalence of antiGAD and ICA islet antibodies (7.3%) amongst Type 2 DM patients in Tanzania.

Majority of the subjects with LADA in this study were in 50–59 age category and less than 5% were in the 30–39 age category. This observation suggests that LADA increases with increasing age decade, confirming reports by [Carlson et al 29] that older age was an important risk factor for LADA as for Type 2 DM and this may suggest a potential role for insulin resistance in the pathogenesis of LADA. A Chinese report also noted that the prevalence of LADA slowly increased with age up to 60 years and was high in individuals aged 50–59 years [9]. [Horton et al 30] reported that genetic influences were determinants of age at presentation in LADA specifically the DRB1/DQB1 genotype with younger LADA subjects demonstrating this genotype while little relation to a specific genotype occurred after the age of 55 years.

Our findings lend credence to previous reports which suggest that the presence of LADA is non sex specific [9]. We have documented in this report that although a higher percentage (64%) of subjects positive for antiGAD autoantibodies in this study were females, the prevalence rates were however comparable in both sexes.

Thirty-seven percent of LADA were already on insulin as at the time of the Study for glycaemic control and this may be a reflection of autoimmune destruction of the β cells which has been reported to be present at diagnosis of DM in LADA patients [31]. This documented observation is not surprising given the fact that we uncovered insulinopaenia in 60% of our Study subjects (that were antiGAD autoantibody positive) who were on insulin. The Hoorn study involving 50–74 year old population reported an association between GAD positivity and insulin use in diabetic subjects [32]. In comparison, 19% of GADA negative individuals were on insulin for glycaemic control in this study, the proportion of LADA subjects on insulin was markedly higher.

The occurrence of other autoimmune antibodies with increased frequency of serologic markers of thyroid and adrenal disease in LADA has been well documented in various studies [33-35]. Eleven percent of LADA patients in this study were found to have history of autoimmune thyroid disease, also confirming earlier reports of increased incidence of humoral immunity to other organ specific autoantibodies especially the thyroid gland [36,37]. The Ehime study in Japan which followed up a cohort of LADA subjects noted, that positive thyroid peroxidase antibodies(Abs) and antithyroglobulin Abs contribute to the progression to B-cell failure in LADA [38].

Less than 50% of subjects with LADA had a history of hypertension and about 30% had history of dyslipidemia. The proportion of LADA subjects with metabolic risk factors of overweight/obesity, hypertension, dyslipidaemia, smoking and alcohol ingestion was lower than GADA –ve subjects with these metabolic risk factors. Similar findings have been reported from Ghana, China, Sweden and Australia [10,38-40]. The aforementioned Ghanaian study (10) found that LADA differed from autoantibody negative type 2 DM in hypertension and central obesity which occurred more frequently in the latter, concluding that generally, clinical and metabolic markers could not be used to differentiate Ghanaian latent autoimmune diabetes from type 2 DM. The proportion of obese/overweight among subjects with LADA in this study was higher than those in normal/underweight category and this suggests insulin resistance as possible contributory factor in the pathogenesis of LADA amongst our patients.

About half of LADA subjects in this study were insulinopenic. Although patients with LADA share insulin resistance with type 2 diabetes patients, they however display a more severe defect in stimulated beta-cell capacity than patients with classical type 2 diabetes. This observation suggests that C-peptide levels could be used as a clinical screening tool in identifying LADA. [Bell and Ovale 41] in a study to assess the effectiveness of random C-peptide levels as screening test for LADA noted that LADA can be ruled out in adult-onset diabetes by the presence of elevated C-peptide.

A higher proportion of subjects with LADA had evidence of microvascular complications of DM namely retinopathy and neuropathy and a higher proportion of GADA –ve individuals manifested nephropathy. The prevalence of dyslipidemia and the metabolic syndrome was also higher in the GADA-ve group. The foregoing would suggest that macrovascular complications of DM were more frequent in GADA-ve patients, while LADA subjects manifest predominantly microvascular complications especially retinopathy. The poor indices of long term glycemic control in LADA may account for the observed higher percentage of LADA subjects with evidence of microvascular DM complications. [Myhill et al 42] noted that subjects with LADA compared with the GAD antibody-negative patients, had a similar prevalence and incidence of coronary heart, cerebrovascular disease, cardiac disease and all-cause mortality. The significant proportion of LADA with retinopathy observed in this study is similar to reports from Turkey [43]. Reports from Western Finland [9] however noted similar prevalence of retinopathy, cardiovascular morbidity and mortality between LADA and GADA-ve subjects and glycaemic control was noted to be a stronger risk factor for cardiovascular disease in LADA subjects.

## Conclusion

We conclude that the prevalence of GAD autoantibody positivity amongst our patients is comparable to what obtains in Ghanaian and Caucasian populations. Proper classification of DM in our setting is thus of necessity.

## Competing interests

The authors declare that they have no competing of interests.

## Authors’ contribution

OOA conceived of the study, was responsible for the design and coordination of study, drafting the manuscript and statistical analysis. AOO was involved in drafting the manuscript, revising manuscript critically for important intellectual content, statistical analysis. OF revised manuscript critically for important intellectual content. OOO participated in the design of study and statistical analysis. AOD analyzed and interpreted the data. AOA participated in the acquisition of data. FMA carried out immunoassays. All authors read and approved the final manuscript.
